# A cross-sectional study of sleep and depression in a rheumatoid arthritis population

**DOI:** 10.1007/s10067-020-05414-8

**Published:** 2020-09-24

**Authors:** Mark Hughes, Alan Chalk, Poonam Sharma, Sandeep Dahiya, James Galloway

**Affiliations:** 1 Rheumatology Department, Kings College London, Denmark Hill, London, UK; 2 Cass Business School, London, UK; 3 Rheumatology Department, Peterborough City Hospital, Peterborough, UK

**Keywords:** Depression, Observational study, Outcomes, Rheumatoid arthritis, Sleep quality

## Abstract

**Objectives:**

To assess the prevalence of impaired sleep quality and depression in a rheumatoid arthritis population and determine their correlation with Disease Activity Score (DAS) and its components.

**Methods:**

In this single-centre observational cross-sectional study, data was collected by the assessing clinician for DAS28, age and gender in various treatment groups according to use of csDMARDs, biologics and long-term steroids. Presence of impaired sleep quality and depression was assessed by the Pittsburgh Sleep Quality Index (PSQI) and Public Health Questionnaire 9 (PHQ 9). Correlation for DAS and its components with the outcomes was determined by Pearson’s correlation coefficient. Multivariate analysis was performed by logistic regression.

**Results:**

Two hundred patients were included. The prevalence across all subgroups of poor sleep quality and depression were 86.5% and 30%, respectively, with a correlation coefficient of 0.69 between the two and poor sleep quality amongst all RA patients with comorbid depression. Multivariate analysis found only subjective DAS components, tender joint count (TJC) and patient global health visual analogue score (VAS) to significantly correlate with both outcomes. Age inversely correlated with depression. Long-term steroid use was associated with poorer sleep quality, but there was no significant effect of csDMARDs or biologics. There was no significant difference in prevalence of depression amongst treatment subgroups.

**Conclusion:**

Poor sleep quality and to a lesser extent depression are prevalent in the general rheumatoid arthritis population. Patients would benefit from clinicians measuring these outcomes routinely as they constitute a significant non-inflammatory burden of living with rheumatoid disease.

**Table:** 

**Key Points** *• Subjective components of DAS independently correlate with sleep quality and depression, while objective components do not.* *• Poor sleep quality is highly prevalent in RA and present in all those with comorbid depression.* *• Poor sleep quality and depression incidence in RA are much lower when DAS is low or remission.*

## Introduction

Rheumatoid arthritis (RA) is known to have a wide range of effects beyond the joints, with established impact on sleep and mood [1]. The cause of impaired sleep quality and depression in RA is not clearly understood. In the general population, multiple episodes of inflammation are predictive of an increased risk of developing depression [2]. Compared with the general population, there is a higher prevalence of depression amongst arthritis patients [3]. Comorbid depression, in multiple physical health conditions including arthritis, is associated with reduced quality of life, overall health [3] and specifically reduced response to biological therapy in RA [4].

In RA, auto-immune synovitis leads to the generation of inflammatory cytokines such as Il-6 that cross the blood-brain barrier and interact with the central nervous system [5]. However tocilizumab, an Il-6 antagonist, has been found to not significantly reduce depression despite significantly improving disease activity [6], implying that other mechanisms are involved beyond the Il-6 axis.

Sleep and depression are strongly linked, with multiple proposed mechanisms of bidirectional causality between the two [7]. Poor sleep quality has been demonstrated to lead to altered mood with both increased intensity of negative moods such as depression and reduced levels of happiness [8]. The presence of mood disturbance has been shown to significantly increase the risk of subsequently developing poor sleep quality over the next year [9].

This study sets out to use cross-sectional data to determine the relationship between RA disease activity with sleep quality and depression: the Sleep QUality And Depression in Rheumatoid ObservatioN (SQUADRON) study.

## Method

This was a single-centre, cross-sectional study, conducted in an English secondary care hospital in June–October 2018. Inclusion criteria were age over 18, consultant diagnosis of RA and seropositive status for RF or ACPA. Exclusion criteria were non-fluency in English and cognitive impairment severe enough to impair ability to consent or understand the questionnaires. All patients meeting inclusion criteria, scheduled to attend rheumatology clinics at the study site during the data collection period, were sent a written invite to participate in the study.

On the day of their consultation, consent was sought to partake, and if given, firstly ESR-based 28 joint count Disease Activity Score (DAS28-ESR) was measured and recorded by the treating clinician. This includes assessment of tender joint count (TJC), swollen joint count (SJC), patient global health visual analogue score (VAS) and ESR blood test. An ESR collected within 1 month of clinician assessment was required. Questionnaires were then given to the patients to complete after the consultation. The questionnaires were rejected if not fully completed.

Sleep quality was assessed by the Pittsburgh Sleep Quality Index (PSQI) questionnaire, which uses 19 questions to determine a score from 0 to 3 for seven components of sleep quality. The PSQI has a maximum score of 21, with higher scores indicating worse sleep. A total score of ≥ 6 was considered a positive result for poor sleep quality [10].

Depression was assessed by Patient Health Questionnaire 9 (PHQ9), a 9-item questionnaire with each question scoring 0–3. PHQ scores range from 0 to 27, with higher scores indicating lower mood. A total score of 10 or greater was deemed to be a positive result for depression [11].

The confounder variables that were assessed were the patient age and gender. They were also categorised according to presence or absence of csDMARDs treatment, presence or absence of biological/nonconventional DMARDs and the use of long-term prednisolone, defined as any oral dose taken for a duration greater than 3 months.

A sample size calculation was performed accepting an *α* of 0.05 and power of 0.8, allowing for four independent variables (DAS components) and two dependent variables (sleep quality and depression). This concluded to reject the null hypothesis if there was a true correlation > 0.25; 200 participants would be required.

Statistical analysis was performed using R software, version 3.4.3. Baseline demographics were presented using descriptive methods with mean and standard deviation. Relationships between dependent and independent variables were tested using Pearson’s correlation coefficients and represented graphically by scatter graphs with Loess curves and 95% confidence interval mapping. Multivariate logistic regression was performed to describe relationships between sleep and depression, incorporating all DAS components, use of csDMARDs/biologics/long-term steroids, age and gender. For smaller subgroup analyses, such as drug therapy, logistic regression via boot strap resampling was used, with adjustment for non-modifiable characteristics such as age and gender. To further evaluate the relationship between sleep and disease activity, we used a linear mixed effects model incorporating depression.

Ethical approval from the National Research Ethics Service was obtained (Ref: 18/WM/0137).

## Results

A total of 535 invites to participate in the study were sent between May and October 2018. The data collection ceased once 200 usable responses were collected, giving a 37.4% response rate overall.

A total of five participants, who had consented to take part, were excluded, two due to missing questions on their PHQ-9 or PSQI questionnaires, two due to no valid ESR sample being available and one due to tender joint count not being documented by the assessing physician.

Patient characteristics and current treatment are documented in Table 1. Age ranged from 21 to 87 years old. Most patients were taking a csDMARD and approximately half were on biologic therapy. A relatively low proportion (13.5%) was on long-term oral steroids.

**Table 1:** General and treatment characteristics of the participants

Age (years), mean (SD)	61.8 (13.9)
Female *n*( %)	147 (73.5)
Current csDMARD use *n* (%)	145 (72.5)
Current biologic DMARD use *n* (%)	91 (45.5)
Current use of long-term oral steroids *n* (%)	27 (13.5)
TJC, mean (SD)	3.5 (5.7)
SJC, mean (SD)	1.2 (2.7)
ESR, mean (SD)	17.6 (18.2)
VAS, mean (SD)	32.4 (25.0)
DAS28-ESR, mean (SD)	3.0 (1.6)
DAS28-ESR < 2.6 *n* (%)	98 (49)
DAS28-ESR 2.6–3.2 *n* (%)	22 (11)
DAS28-ESR 3.2–5.1 *n* (%)	56 (28)
DAS28-ESR >5.1 *n* (%)	24 (12)
PSQI score, mean (SD)	9.6 (3.7)
PHQ-9 score, mean (SD)	6.9 (6.2)

The PHQ9 scores were positively skewed, with a mean of 6.92 and a standard deviation of 6.191; 60 (30%) of respondents scored greater than or equal to 10 indicative of depression. Fifteen participants (7.5%) expressed a degree of suicidal ideation as question 9 of PHQ9.

There was a significant interrelationship between PSQI and PHQ9, with a correlation coefficient of 0.69 (95% CI 0.60–0.75) (*p* < 0.001)**.** There was poor sleep quality amongst all those who were depressed and 81% prevalence of poor sleep quality amongst those not depressed, giving a prevalence of 86.5% overall.

Table 2 demonstrates the incidence of depression and poor sleep quality, with subgroup analysis according to disease activity level. Good sleep quality was present in 13.5% of patients overall. Of the patients in remission, 23.5% had good sleep quality and in 2%, 1% and 1% for patients with low, moderate and high disease activity, respectively. Depressive symptoms were present just over 50% of patients in both moderate and severe disease activity but progressively diminished in those with low activity or remission.

**Table 2:** Numbers of patients demonstrating good vs poor sleep and depression vs no depression

Total *n* = 200	Good sleep quality (27)	Poor sleep quality (173)
Not depressed (%)	27 (13.5)	113 (56.5)
Depressed (%)	0 (0)	60 (30)
Remission, DAS < 2.6 *n* = 98		
Not depressed (%)	23(23.5)	63(64.3)
Depressed (%)	0 (0)	12 (12.2)
Low activity, DAS 2.6–3.2 *n* = 22		
Not depressed (%)	2(9.1)	15 (68.2)
Depressed (%)	0 (0)	5 (22.7)
Moderate activity, DAS 3.2–5.1 *n* = 56		
Not depressed (%)	1(1.8)	25 (44.6)
Depressed (%)	0 (0)	30 (53.6)
Severe activity, DAS > 5.1 *n* = 24		
Not depressed (%)	1 (4.2)	10 (41.7)
Depressed (%)	0 (0)	13 (54.2)

### DAS components

**Fig. 1:**
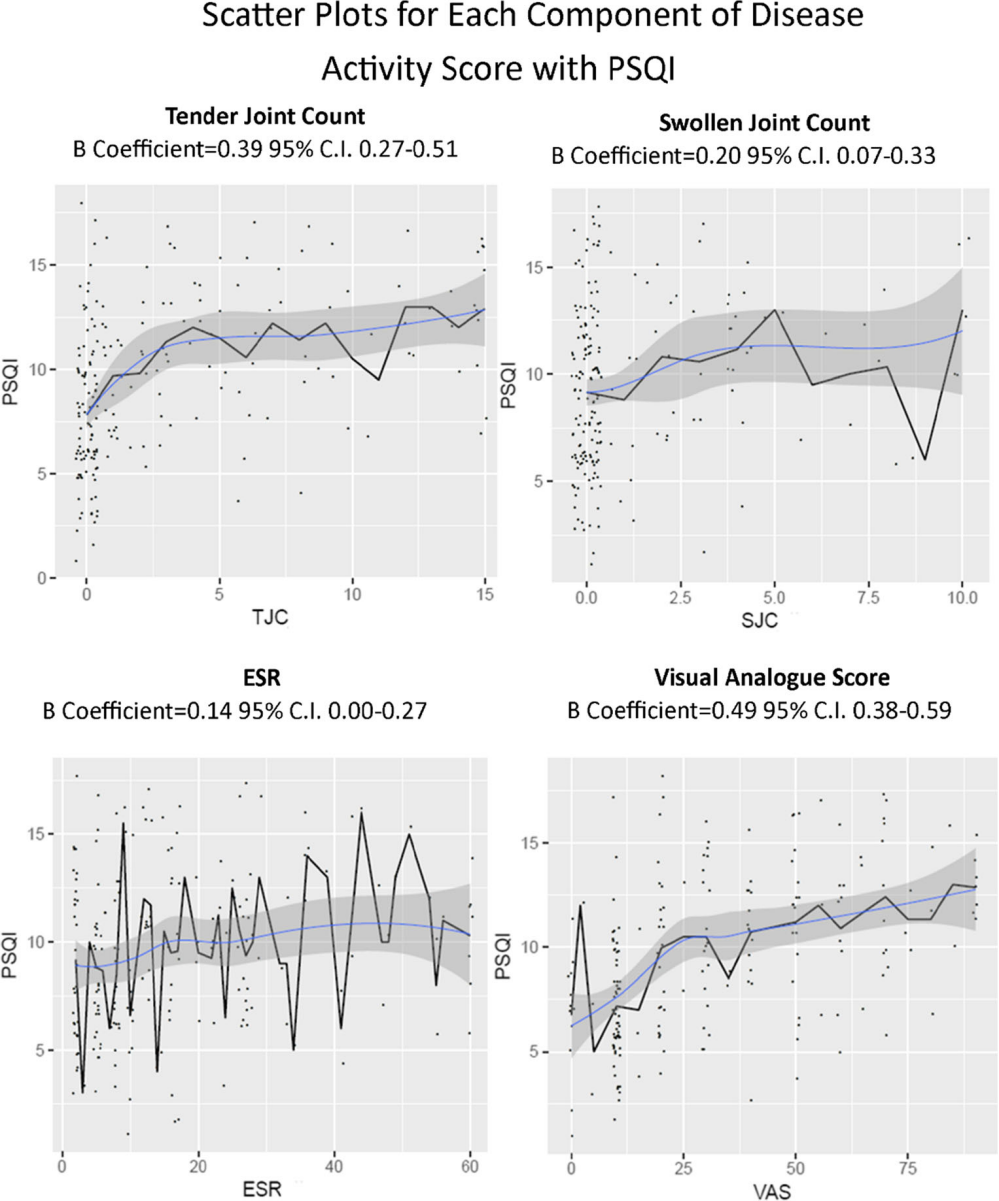
Scatter plots for each component of disease activity score with PSQI

**Fig. 2:**
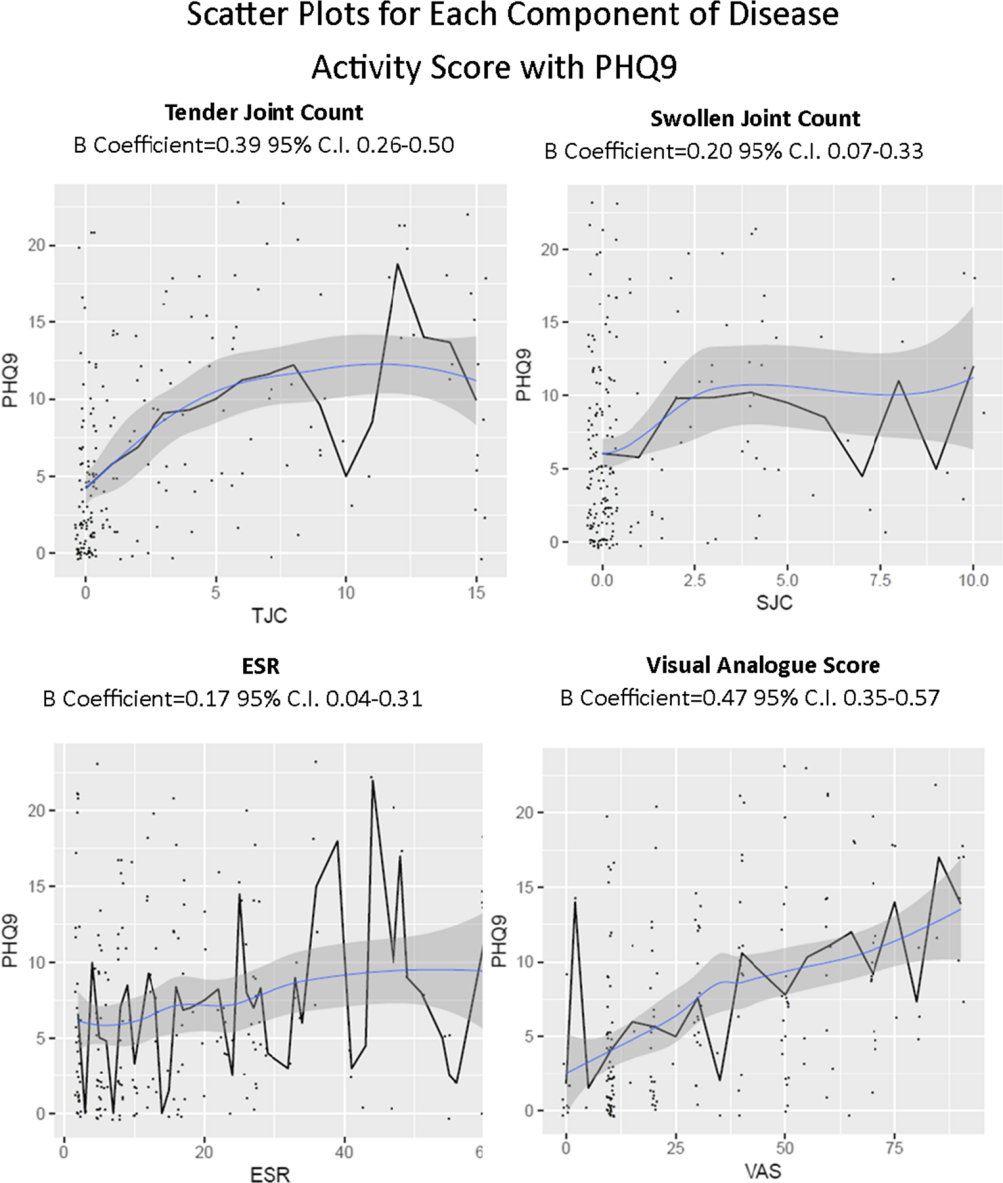
Scatter plots for each component of disease activity score with PHQ9

The relationships between the four components of DAS with PSQI and PHQ9 are demonstrated in Figs. 1 and 2, respectively. Each dot represents one respondent, with the black line representing average response and the blue line representing a Loess curve with the surrounding grey area for 95% confidence interval.

All components demonstrated very similar correlation coefficients for both PSQI and PHQ9. ESR and SJC both demonstrated relatively weak correlation, with TJC and VAS demonstrating higher beta coefficients.

### Drug therapy

There was no significant difference in PSQI score according to whether patients were on csDMARDs (odds ratio (OR) 1.4 (*p* = 0.560, 95% CI 0.5–3.8)) or biologics (OR 0.9 (*p* = 0.748, 95% CI 0.3–2.2)); this remained the case with subgroup analysis of depressed and non-depressed cases. The number taking long-term steroids was relatively small for subgroup analysis (27), but with boot strap re-sampling to account for the small sample size, the odds ratio for poor sleep quality when taking long-term steroids was 4.6 (95% CI 0.6–35.4), which was not significant. After adjusting for age and gender, this increased to 5.3 (95% CI 1.7–16.8, *p* < 0.005). There were no significant associations between csDMARDs (OR 0.7 (*p* = 0.255, 95% CI 0.3–1.3)), biologics (OR 0.9 (*p* = 0.772, 95% CI 0.4–1.8)) or steroid use (OR 2.1 (*p* = 0.093, 95% CI 0.9–5.0)) and depression.

### Multivariate analysis

To further assess the correlates with PSQI and PHQ9 score, all 4 components of DAS were entered into multivariate logistic regression. Age, gender, use of csDMARDs, use of biologic DMARDs and use of long-term steroids were also entered into the analysis.

For PSQI the only variables to meet statistical significance on multivariate logistic regression were TJC and VAS, with an increase in PSQI by 0.293 standard deviations (SD) for every increase TJC by 1 standard deviation and 0.461 for every VAS SD. The correlation between TJC and PSQI was non-linear, and there was significantly greater correlation between the square root of TJC and PSQI than TJC, increasing to 0.341. When correcting for TJC and VAS, SJC and ESR did not influence PSQI.

For PHQ9, multivariate logistic regression revealed age to be negatively correlated with PHQ9 score reducing by 0.197 SD for every increase in age by 1 SD, meaning a reduction in PHQ9 by 0.83 for every decade change in age. TJC and VAS were positively correlated, increasing PHQ9 by 0.149 SD and 0.363 SD, respectively, for every increase by 1 SD. Again the correlation was greater for square root of TJC than TJC, increasing to 0.257, and similarly when correcting for TJC and VAS, SJC and ESR did not influence the result.

**Fig. 3:**
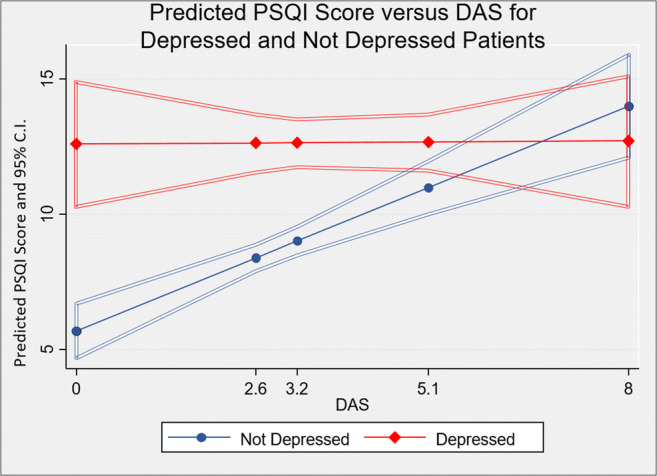
Predicted PSQI Score versus DAS for depressed and not depressed patients

In the mixed effects model, people without depression showed a positive correlation between sleep and disease activity. However, amongst individuals with depressive symptoms, no relationship was apparent. Figure 3 shows these findings, depicting predicted PSQI values based on the multivariate logistic regression comparing those with and without depression.

## Discussion

The results of this study demonstrate that the majority (86.5%) of RA patients experience poor sleep quality, which is highly correlated with depression. The prevalence of sleep disturbance amongst RA patients attending outpatient appointments in this study population was higher than most other studies assessing PSQI score in RA populations. Sariyildiz [12] found a much lower proportion (64%) of RA patients to have poor sleep quality. However, the population was less representative in this study as patients with chronic medical or psychiatric comorbidities were excluded, which likely explains the much lower incidence of poor sleep quality.

The subjective components of DAS, TJC and VAS, both significantly correlate with PSQI and PHQ-9, whilst the objective components, SJC and ESR, do not. Treatment strategies do not significantly correlate with either outcome measure, other than an increase in PSQI amongst those on long-term steroids, but the group size was relatively small.

Inflammatory cytokines, particularly TNF-alpha and IL-6, have been implicated in the pathogenesis of psychiatric illnesses including major depression [13]. The results from SQUADRON multivariate logistic regression to some extent undermine this theory, perhaps suggesting that the direct role of inflammation in depression may be relatively limited, given the absence of correlation with objective measures of disease activity.

A possible explanation would be that altered pain perception, i.e. ‘secondary fibromyalgia’, rather than disease activity or inflammation is the main determinant of sleep disturbance. This would corroborate previous findings of Lee [14], who found that RA patients have increased central pain amplification compared with controls, the effect of which is likely mediated by sleep disturbance. Pollard [15] demonstrated that reduced pain threshold in RA patients strongly correlated with subjective DAS components but not the objective measures indicating that reduced pain threshold was a consequence of central sensitisation or ‘secondary fibromyalgia’ rather than active inflammation; this also appears to be the case for sleep disturbance in our studied population. The reported prevalence of comorbid fibromyalgia in rheumatoid arthritis patients varies greatly, from 4.9 [16] to 52.4% [17]. A recent meta-analysis found a pooled effect of 21% (95% confidence internal 17–25% [18].

The only other factor that Pollard [15] found to correlate with reduced pain threshold was disease duration. This raises the possibility that a degree of long-term structural damage to joints rather than current RA activity could contribute to degree of altered pain processing, mood disturbance and impaired sleep quality. This could account for the lack of demonstrable benefit from biologic treatment. Whilst most of our cohort had well-controlled disease at the time of the study, they will by definition have suffered severely active disease in the past in order to qualify for a biologic. An alternative explanation would be that those who have increased central pain amplification are more likely to meet the criteria for commencing biologic therapy (DAS > 5.1 in the UK).

Corticosteroids are known to cause difficulty initiating and maintaining sleep [19], which is consistent with our finding of significantly higher PSQI scores amongst those on long-term steroids. This supports the EULAR guidelines advising against long-term glucocorticoids in the management of RA [20].

The strengths of this study are firstly that it was carried out in a real-world clinic setting, with minimal selection of participants other than adequate English comprehension, making the findings widely generalisable. Secondly the size of the sample is one of the largest observational studies for sleep quality and/or depression in RA patients.

The limitations to consider are the cross-sectional design, so caution must be employed regarding causal inferences. Selection bias cannot completely be ruled out due to the study design. The outcome measures are self-reported and cannot be interpreted as truly objective measures of sleep quality. Two of the questions on PHQ 9 relate to sleep or fatigue which may lead to some duplication, increasing the correlation between the two outcomes. It is important to acknowledge that there is potential unmeasured confounding present, for example, relating to aspects such as comorbidities which were not captured in the study. Finally, a recommendation for future research into sleep in RA would be to prospectively capture diagnostic information on fibromyalgia as this comorbidity is closely linked.

Awareness of comorbid depression in RA patients may help to avoid inappropriate treatment escalation. Other treatments such as cognitive behavioural therapy or initiation of anti-depressant therapy could both potentially be initiated in rheumatology clinics, with more severe cases referred to psychiatric services. Impaired sleep quality in inflammatory arthritis may be firstly addressed by discussing sleep hygiene as per the EULAR guidelines [21], as well as screening for obstructive sleep apnoea and considering referral to a sleep specialist if appropriate. More recent studies have shown that exercise, in addition to numerous other benefits, may help to improve sleep disturbance in RA patients [22].

In conclusion, poor sleep is a common problem in people with RA. It is highly correlated with concomitant low mood. Given that these disorders are rated as some of the most important extra-articular symptoms of RA by patients, assessing them should be part of routine care. Significant sleep disturbance and depression persists across the disease activity spectrum until a state of low disease activity at a minimum is reached, highlighting the need to treat to a target of low disease activity or remission.
